# Association between cystic fibrosis transmembrane regulator genotype and clinical outcomes, glucose homeostasis indices and CF-related diabetes risk in adults with CF

**DOI:** 10.1590/1678-4685-GMB-2023-0021

**Published:** 2024-03-29

**Authors:** Noémie Bélanger, Anne Bonhoure, Tamizan Kherani, Valérie Boudreau, François Tremblay, Annick Lavoie, Maite Carricart, Ashish Marwaha, Rémi Rabasa-Lhoret, Kathryn J. Potter

**Affiliations:** 1 Montreal Clinical Research Institute, Montreal, QC, Canada.; 2 Université de Montréal, Faculté de Médecine, Département de Nutrition, Montréal, QC, Canada.; 3 University of Alberta, Department of Pediatrics, Division of Pediatric Respirology, Edmonton, AB, Canada.; 4 Université de Montréal, Faculté de Médecine, Département de Médecine, Montréal, QC, Canada.; 5 Centre Hospitalier Universitaire de Montréal, Clinique de Fibrose Kystique, Montréal, QC, Canada.; 6 University of Calgary, Cumming School of Medicine, Department of Medical Genetics, Calgary, AB, Canada.

**Keywords:** CFTR variants, Cystic Fibrosis, Oral Glucose Tolerance Test, L206W, A455E

## Abstract

People living with cystic fibrosis (pwCF) homozygous for F508del present more severe phenotypes. PwCF with compound heterozygous genotypes F508del /A455E and F508del /L206W may have milder cystic fibrosis (CF) phenotypes. We compared F508del homozygotes and common compound heterozygotes (F508del and a second pathogenic variant) in adult patients. Nutritional, pulmonary function and glucose homeostasis indices data were collected from the prospective Montreal CF cohort. Two-hundred and three adults with CF having at least one F508del variant were included. Individuals were divided into subgroups: homozygous F508del/F508del (n=149); F508del/621+1G>T (n=17); F508del/711+1G>T (n=11); F508del/A455E (n=12); and F508del/L206W (n=14). Subgroups with the F508del/L206W and F508del/A455E had a lower proportion with pancreatic exocrine insufficiency (p<0.0001), a higher fat mass (p<0.0001), and lower glucose area under the curve (AUC) (p=0.027). The F508del/L206W subgroup had significantly higher insulin secretion (AUC; p=0.027) and body mass index (p<0.001). Pulmonary function (FEV1) was significantly higher for the F508del/L206W subgroup (p<0.0001). Over a median of 7.37 years, the risk of developing CFRD in 141 patients was similar between groups. PwCF with heterozygous F508del/L206W and F508del/A455E tended to have pancreatic exocrine sufficiency, better nutritional status, improved pulmonary function and better diabetogenic indices, but this does not translate into lower risk of CF-related Diabetes.

## Introduction

Cystic fibrosis (CF) is an autosomal recessive condition caused by pathogenic variants in the regulatory regions of the CF transmembrane conductance regulator (CFTR) (Iafusco et al., 2021). CFTR encodes a transmembrane chloride channel that is critically important to electrolyte composition of mucoid secretions in multiple organ systems (Desgeorges et al., 1995). Individuals with CF most commonly experience respiratory, digestive and gastrointestinal, and endocrine complications (Iafusco *et al.,* 2021). Nowadays, cystic fibrosis-related diabetes (CFRD) is the most frequent non-pulmonary complication of CF.

There are over 2000 variants in the CFTR gene, with over 700 being described as pathogenic, and therefore leading to CF or related phenotypes, according to the CFTR2 Database (updated variant list April 7, 2023) (Mckone et al., 2003; Castellani and Assael, 2017). At first appearance, the HVGS name will be used followed by the legacy name in parentheses. Afterwards, only the legacy name will be used throughout the rest of the manuscript. Most individuals with CF will have at least one c.1521_1523delCTT (F508del) pathogenic variant detected (Alfonso-Sanchez et al., 2010). In 2019, approximately 47.1% of Canadian adults with CF were homozygous F508del, 40.7% were heterozygous (F508del and another allele) and 12.3% had another genotype. F508del is a class II pathogenic variant, in which there is defective protein processing (Mckone *et al.,* 2003). Individuals homozygous for F508del typically present with earlier and more severe respiratory complications and have earlier mortality (Johansen et al., 1991; De Braekeleer et al., 1997a; Mackenzie et al., 2014; Keogh et al., 2018). Most F508del homozygotes have exocrine pancreatic insufficiency but the effect of this genotype on weight and body mass index is controversial (Lanng et al., 1991; Santos and Steemburgo, 2015; Leung et al., 2020; Medza et al., 2021). Homozygotes for F508del also have impaired glucose tolerance and a higher risk of cystic fibrosis-related diabetes (CFRD) (Hamdi et al., 1993; Street et al., 2012). The variants c.1364C>A (A455E) and c.617T>G (L206W) in trans with F508del have been associated with milder phenotypes (Hamosh and Corey, 1993; Desgeorges et al., 1995; Gan et al., 1995; Rozen et al., 1995; De Braekeleer *et al.,* 1997a; Mckone *et al.,* 2003). 

The Montreal CF cohort (MCFC), established in 2004, prospectively follows over 300 individuals with CF, which allows for the study of nutritional and diabetogenic status over time. Given the predominantly francophone origins of the cohort and a common founder population in Quebec, we compared the phenotype of F508del homozygotes with those of compound heterozygotes (F508del with another pathogenic variant) with a focus on *CFTR* variants that are common in the French-Canadian population. Some *CFTR* variants, mostly belonging to the IV and V pathogenic variant classes, lead to a milder CF phenotype (Gan et al., 1995; Rozen et al., 1995; De Braekeleer et al., 1997a ; Clain et al., 2005; Castellani and Assael, 2017). Previous genotype-phenotype correlations amongst the French-Canadian population have identified that amongst the F508del compound heterozygotes, the most common variants in trans with F508del were c.579+1G>T (711+1G>T) (9%), c.489+1G>T (621+1G>T) (5%), A455E (1%), and L206W (1%), however, the most frequent is another F508del variant (71%) (Rozen *et al.,* 1992 *,* 1995). Genotype-phenotype correlations have been characterized in small case studies, many of which were documented in the early 1990s (Rozen *et al.,* 1992, 1995; Desgeorges et al., 1995; Gan *et al.,* 1995; Witt et al., 1996; De Braekeleer *et al.,* 1997a, b, c). 

The objective of this study was to compare the clinical and diabetogenic phenotype of F508del homozygotes and compound heterozygotes (F508del and a second pathogenic variant) in the MCFC. In particular, we wanted to examine the risk of developing CFRD according to genotype.

## Material and Methods

A total of 203 individuals with CF from the MCFC had available data from their baseline visit. All data related to CFTR genotype were extracted from the Canadian Cystic Fibrosis Registry (2019). Measurements were all taken at the Hospital for Sick Children in Toronto (Petruzziello-Pellegrini et al., 2021). Clinical data and oral glucose tolerance testing data (including serum glucose and insulin at fasted (T0) and 30 (T30), 60 (T60), 90 (T90) and 120 (T120) minutes post-glucose challenge) were collected for each individual, as previously described using a standardized glucose load (1.75 g/kg, to a maximum of 75g) (Boudreau et al. 2016). Individuals were classified into glucose tolerance categories as follows: normal glucose tolerance [NGT; G0: <7.0mmol/L and G120: <7.8mmol/L], impaired glucose tolerance [IGT; G0: <7.0mmol/L and G120: ≥7.8 and <11.1mmol/L], indeterminate glucose tolerance [INDET; G0: <7.0mmol/L and G120: <7.8, but G60: ≥11.1mmol/L], and CFRD (G0<7.0mmol/L and G120: ≥11.1mmol/L) (Moran et al., 2010). Pancreatic insufficiency was recorded in terms of enzymes intake, which are prescribed based on a fecal elastase 1 dosage test (extracted from medical records). None of the individuals with CF and pancreatic sufficiency were on pancreatic enzymes. The percentage of fat was obtained by impedance measurement on an electronic scale (Tanita Corporation Arlington Heights, IL, USA). Detailed data collection and procedures were already described (Boudreau *et al.,* 2019).

A total of 141 patients with at least one follow-up visit with complete glucose and insulin data were included in the prospective study. Kaplan-Meier survival analysis was restricted to individuals with 1+ follow-up visits and with complete glucose and insulin data. 

Statistical analyses were performed on SPSS software (IBM, version 26). Descriptive statistics were computed for all variables of interest. Mean ± SD was used to present data. Assumptions of normality were checked. Then, for continuous variables, parametric (one-way ANOVAs) or non-parametric tests (Kruskall-Wallis) were used accordingly to compare between the genotype subgroups. For categorical variables, such as sex and pancreatic enzyme intake, χ^2^ logistic regression was performed using absolute frequencies. Finally, to assess the risk of developing CFRD, OGTT data of subsequent visits were collected and a Kaplan-Meier survival analysis comparing the genotype subgroups was performed using GraphPad Prism (GraphPad Software, USA). The Mantel-Cox test was used to calculate a *p* value for the Kaplan-Meier analysis. A *p* value ≤0.05 implied significance for all analyses. All *p* values are determined by ANOVAs unless specified otherwise. 

The protocol for the MCFC (cohort established to study the mechanisms leading to CFRD and its screening) was approved by the Centre Hospitalier de l’Université de Montréal (CHUM) research ethics committee, and all participants signed an information and consent form (protocol #MP-02-2004-1717).

## Results

### Description of the cohort

Of the total 308 individuals in the MCFC, we selected 203 individuals (66% of cohort) who had at least one F508del variant or whose variants were documented. Of this group, 73% (n=149) were homozygous for F508del and 27% (n=54) were compound heterozygotes. For all individuals, the mean age (years ± SD) at baseline was 25.36 ± 7.72. The mean weight was 59.12 ± 10.72 kg while the mean body mass index (BMI) was 21.43 ± 2.97 kg/m^2^. Fat mass was 18.09 ± 7.73% and pulmonary function expressed by the predicted (%) forced expiratory volume in 1 second (FEV_1_) was 69.21 ± 21.65%.

We selected F508del compound heterozygotes to compare to F508del homozygotes if there were at least 10 patients with this genotype. Compound heterozygote groups with fewer than 10 subjects were excluded from analysis. The groups included in analysis were as follows: F508del/F508del (n=149, 73.4%), F508del/621+1G>T (n=17, 8.4%), F508del/711+1G>T (n=11, 5.4%), F508del/A455E (n=12, 5.9%), and F508del/L206W (n=14, 6.9%). 

**Figure 1 -:**
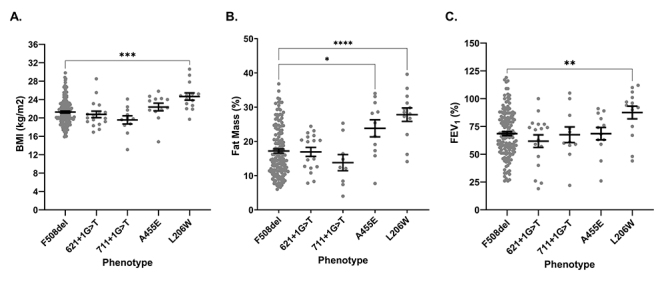
Clinical markers of nutritional status and pulmonary function are significantly higher in the F508del/L206W subgroup. Body mass index (kg/m^2^) (A), fat mass (%) (B), and FEV1 (%) (C) were collected at baseline and analyzed as independent events according to the second variant. Data are shown as mean ± standard error of the mean. Significance was determined using one-way ANOVAs and is demonstrated as follows: *: p < 0.05; **: p < 0.01; ***: p < 0.001; ****: p < 0.0001

In Table 1, we compared the clinical phenotype of each subgroup. Weight and BMI in the F508del/L206W subgroup were significantly higher than all other subgroups except for F508del/A455E (p=0.007 and p<0.001, respectively). Fat mass was higher for both F508del/L206W and F508del/A455E subgroups (p<0.001) while FEV_1_ was significantly higher for F508del/L206W (p=0.014). Visual representation according to the second variant in trans with F508del can be found in Figure 1 for BMI (A), fat mass (B), and FEV_1_ (C).

**Table 1 -:** Characteristics (± SD) of individuals at baseline according to the genotype.

	Homozygous F508del / F508del	F508del / 621+1G>T	F508del / 711+1G>T	F508del / A455E	F508del / L206W	***P* value** ^‡^
Number of individuals, n	149	17	11	12	14	
Age (years)	24.14 ± 6.35	25.13 ± 6.23	22.91 ± 5.38	30.25 ± 7.96	36.14 ± 13.02	**<0.001^ǂ^**
Gender, male (%)	61.1	47.1	63.6	25.0	35.7	0.052*
Weight (kg)	58.99 ± 10.59	55.35 ± 9.35	54.27 ± 6.96	60.37 ± 10.54	67.81 ± 12.22	**0.007**
BMI^†^ (kg/m^2^)	21.27 ± 2.78	20.76 ± 2.92	19.56 ± 2.95	22.37 ± 2.91	24.65 ± 2.96	**<0.001**
FEV_1_^†^ (%)	68.51 ± 20.91	61.76 ± 23.31	67.55 ± 23.33	68.50 ± 19.29	87.50 ± 21.34	**0.014**
Number of pulmonary exacerbations in the last year	5.99 ± 14.55	8.06 ± 11.32	6.27 ± 9.17	8.08 ± 20.27	1.50 ± 5.61	0.724
Fat mass (%)	17.19 ± 7.23	16.92 ± 5.44	13.79 ± 7.08	23.82 ± 8.27	27.81 ± 7.09	**<0.001**
% requiring pancreatic enzyme supplementation	97.3	94.1	100.0	25.0	7.1	<0.001*
Fasting glucose (mmol/L)	5.46 ± 0.73	5.64 ± 0.68	5.85 ± 1.31	5.31 ± 0.43	5.05 ± 0.42	0.059
120 min glucose (mmol/L)	8.18 ± 3.33	8.33 ± 3.08	11.51 ± 5.00	6.48 ± 2.28	6.91 ± 1.53	**0.035^ǂ^**
Fasting insulin (µU/mL)	9.78 ± 4.82	9.45 ± 3.55	10.51 ± 2.32	8.56 ± 3.13	11.02 ± 2.22	0.678
120 min insulin (µU/mL)	46.72 ± 31.11	48.53 ± 26.58	43.43 ± 24.36	43.75 ± 20.81	70.33 ± 39.21	0.127^§^
AUC glucose	1144.75 ± 266.09	1154.12 ± 245.03	1348.68 ± 520.59	954.58 ± 127.63	977.69 ± 157.41	**0.015**
AUC insulin	4555.94 ± 2149.81	4820.39 ± 2033.39	4137.55 ± 1667.05	5321.92 ± 2521.71	8275.06 ± 5194.50	**0.005**
AUC insulin/glucose	4.21 ± 2.16	4.43 ± 2.02	3.70 ± 2.21	5.66 ± 2.69	8.57 ± 5.35	**<0.001**
Stumvoll insulin sensitivity index	0.118 ± 0.016	0.119 ± 0.013	0.112 ± 0.021	0.124 ± 0.013	0.114 ± 0.013	0.443

† BMI : Body mass index; FEV_1_: Forced expiratory volume in 1 second; ^‡^ *P* < 0.05 was considered significant; * Chi-square test; ^ǂ^ Kruskall-Wallis test; All other tests were performed using one-way ANOVAs; ^§^ Despite the overall test not being significant, the pairwise comparison between the F508del/L206W and the homozygous F508del subgroups was significant (p=0.011).

Area under the curve (AUC) glucose was significantly lower for both F508del/L206W and F508del/A455E subgroups (p=0.015) while insulin secretion (AUC) was higher for the F508del/L206W subgroup only (p=0.005) (Figure S1). Moreover, individuals in the F508del/L206W and F508del/A455E subgroups were less frequently pancreatic insufficient (p<0.001, χ^2^ test). Glucose tolerance status did not differ according to the variant. Interestingly, no individuals in the F508del/L206W subgroup had *de novo* CFRD at baseline and only 1 individual in the F508del/A455E subgroup had CFRD.

### Risk of developing CFRD

In total, 141 participants were included in the prospective analysis. The mean follow-up was 7.37 years with a maximum of 15.58 years. In Table 2, we showed that there was no difference in the proportion who developed CFRD over the prospective study according to genotype. Interestingly, none of the individuals in the F508del/L206W subgroup developed CFRD in the subsequent visits, but two individuals in the F508del/A455E subgroup developed CFRD. CFRD was the most prevalent in the homozygous F508del subgroup.

**Table 2 -:** Proportion of individuals who previously did not have CFRD at baseline who developed CFRD over 15 years, according to genotype.

	Homozygous F508del / F508del	F508del / 621+1G>T	F508del / 711+1G>T	F508del / A455E	F508del / L206W	***P* value** ^‡^
Proportion who did not have CFRD at baseline who developed CFRD (%)	27.0 (27/100)	15.4 (2/13)	16.7 (1/6)	22.2 (2/9)	0 (0/13)	0.187

‡ *P* < 0.05 was considered significant. Analysis was performed using Chi-square.

**Figure 2 -:**
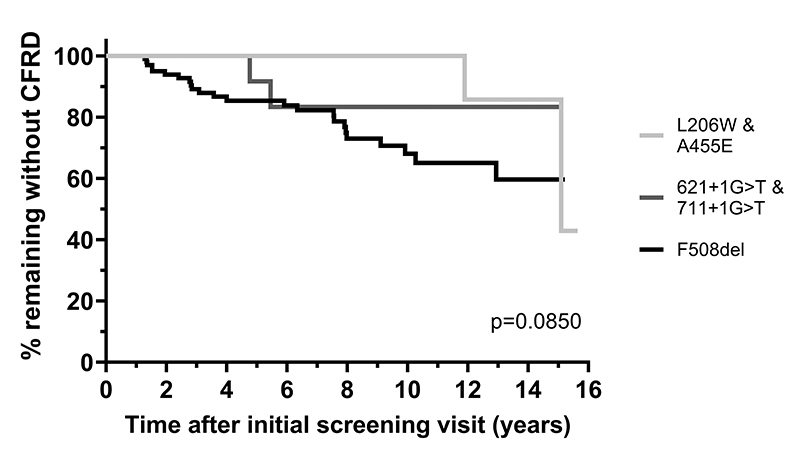
Kaplan-Meier analysis of risk of developing CFRD for common compound heterozygotes (F508del and a second pathogenic variant) as compared to those homozygous for F508del. Homozygous F508del are represented by a black line. F508del/L206W and F508del/A455E compound heterozygotes are represented by a light grey line. F508del/621+1G>T and F508del/711+1G>T compound heterozygotes are represented by a medium grey line. Significance was determined using the log rank (Mantel-Cox) test.

Kaplan-Meier survival analysis of the risk of developing CFRD (Figure 2) showed a trend towards a lower risk of CFRD in those with heterozygous F508del/L206W and F508del/A455E genotypes as compared to those with homozygous F508del (p=0.1294, Mantel-Cox test). 

## Discussion

In this large predominantly francophone cohort, individuals with compound heterozygous F508del/L206W or F508del/A455E CFTR variants 1) tended to have less frequent exocrine pancreatic insufficiency; 2) had preserved pulmonary function; 3) better clinical and nutritional status; and 4) better glucose homeostasis indices which tended to translate into a lower risk of developing CFRD. Other variants when found in trans with F508del such as 711+1G>T and 621+1G>T did not seem to confer milder phenotypes.

### Lung function

Accumulation of mucus and chronic infections cause lung damage and eventually loss of lung function, which appears to be the predominant cause of mortality (Mackenzie et al., 2014; Castellani and Assael, 2017). The F508del/L206W genotype was associated with better lung function as previously reported (Rozen et al., 1995; Clain et al., 2005). In the MCFC, similar results were obtained as the L206W variant was associated with an improved lung function (+19% FEV_1_ for F508del/L206W compared to F508del homozygotes). Literature also suggests a better pulmonary function in F508del/A455E individuals (Gan et al., 1995; De Braekeleer et al., 1997a). In our study, lung function appears to be preserved, but this varies greatly between individuals. For the F508del/621+1G>T and F508del/711+1G>T variants, our results support previous case reports which suggested poorer lung function in these genotypes (Witt et al., 1996; De Braekeleer *et al.,* 1997b *,* c).

### Nutritional and exocrine pancreatic status

In both previous literature (Desgeorges et al., 1995; Clain et al., 2005; Lucarelli et al., 2015) and the CFTR2 database, the L206W variant has been linked to a higher frequency of pancreatic exocrine sufficiency than typically observed in homozygous patients. In the CFTR2 database, which provides information on the different genotypes of over 88 000 patients with CF around the world, 77% of F508del/L206W individuals are pancreatic sufficient. Indeed, in our cohort, 93% of F508del/L206W are pancreatic sufficient compared to only 3% of homozygotes. Accordingly, nutritional status, which is crucial for CF individuals, was more optimal for individuals presenting F508del/L206W variant with higher BMI (+3 kg/m^2^), weight (+9kg) and fat mass (+10%) than F508del homozygotes in our study.

Past reports also suggest that the A455E variant was associated with a greater proportion of individuals with pancreatic sufficiency and higher weight in F508del/A455E heterozygous adult and pediatric individuals as compared to F508del homozygotes (Gan et al., 1995; De Braekeleer et al., 1997a ; Mckone et al., 2003). Our results on 12 F508del/A455E individuals showed that nutritional status was more optimal, as shown by a significantly higher fat mass (over 6% higher than F508del homozygotes). As opposed to previous reports with fewer individuals, weight and BMI did not differ from homozygotes individuals in our study population (Mckone *et al.,* 2003). The proportion of F508del/A455E individuals taking pancreatic enzymes was 25%, possibly indicating a preserved pancreatic function, as found in the Netherlands and in another Québec report (Gan *et al.,* 1995; De Braekeleer *et al.,* 1997a). Once again, the CFTR2 database also predicts pancreatic sufficiency in such individuals as 36 out of 110 patients (33%) are pancreatic insufficient.

The 621+1G>T variant is associated with a severe phenotype (De Braekeleer et al., 1997b ) including a very high risk for exocrine pancreatic insufficiency. In our study, we obtained similar results with the 621+1G>T variant conferring a similar phenotype to the F508del homozygotes. Little is known about the 711+1G>T variant, despite its prevalence in the French-Canadian population. A study comparing 711+1G>T/F508del heterozygotes with F508del homozygotes did not find any significant differences in terms of nutritional between the two phenotypes (Mckone et al., 2003). Our results appear to support previous literature as no difference was found between F508del homozygous individuals and F508del/711+1G>T individuals. Similar conclusions can also be drawn from the 172 patients included in the CFTR2 database.

### Diabetogenic potential according to phenotype

Some indices for glucose homeostasis tended to be or were more favorable for some heterozygous subgroups. For instance, this was shown with higher insulin secretion indices for F508del/L206W individuals and lower blood glucose 2 hours post-OGTT for F508del/A455E individuals. 

CFRD is a frequent complication of CF. Previous literature has shown that genotypes defined as mild (vs. severe) are associated with a lower risk of developing CFRD (Stecenko and Moran, 2010; Lewis et al., 2015; Olesen et al., 2020). Studies have shown that patients with the F508del variant in the homozygous state have lower peak insulin secretion and insulin sensitivity, as well as are more likely to have impaired glucose metabolism and develop CFRD (Cotellessa et al., 2000; Stecenko and Moran, 2010; Street et al., 2012). Though impaired glucose metabolism is still observed in individuals with the F508del variant in the heterozygous state, the prevalence is 16-38% lower than when in the F508del homozygous state (Street *et al.,* 2012; Iafusco et al., 2021). Our findings show similar results and support these previous studies, though we were able to investigate more closely specific genotypes rather than grouping them into mild vs. severe genotype groups or focusing on F508del homozygosity, which was the case in most of the other studies. 

In terms of CFRD risk, at baseline, three heterozygous subgroups (F508del/L206W, F508del/A455E and F508del/621+1G>T) tended to have higher proportions of individuals presenting normal glucose tolerance and fewer individuals tending to have *de novo* CFRD. During follow-up, heterozygous subgroups also tended to present a lower risk of CFRD development than homozygous patients with a very low risk for some genotypes as none of the individuals presenting F508del/L206W and F508del/A455E genotypes developed CFRD. However, there were no significant differences between groups in the prospective follow-up. 

Severe phenotype (De Braekeleer et al., 1997b), including exocrine pancreatic insufficiency, associated with the 621+1G>T variant (Hamosh and Corey, 1993; Witt et al., 1996; Mckone et al., 2003) are two well-established risk factors for CFRD risk (Coderre et al., 2021; Potter et al., 2021). In our study as well as in another small case report also in French-Canadian patients (De Braekeleer *et al.,* 1997c), this did not translate into a high risk of developing CFRD. Though the 711+1G>T variant was associated with lower insulin sensitivity and insulin secretion, individuals with this genotype did not appear to have increased risk of developing CFRD. These observations, however, are limited by the small sample size.

### Limitations

Our study presented some limitations. First, though we are relying on a large well characterized cohort, this is a single-center study with relatively homogeneous French-Canadian patients and the number of participants in some subgroups was larger than in most previous reports but still limiting the statistic power of some analyses. Second, we did not have data on family history of diabetes of frequency of steroid use, both of which are potential confounding factors in the development of CFRD. Third, as with any observational analysis, causality cannot be established, and lower disease severity and/or risk of developing CFRD may be due to better therapeutic options and/or compliance. Finally, this is the first report with detailed glucose homeostasis analysis for some of these variants. Trends suggest a low risk of dysglycemia for individuals with compound heterozygous F508del/L206W or F508del/A455E CFTR variants than with F508del homozygote patients but larger sample sizes in more diverse patient groups are required to establish if these genotypes could have implications for CFRD risk with implications for screening strategies.

## Conclusion

Our results support the fact that the L206W and the A455E variants, when found in trans with F508del, are associated with milder phenotypes than F508del homozygotes. This translates into better nutritional and clinical status. For the first time we report that these variants are associated with preservation of some glucose homeostasis indices, however, these differences did not translate into a lower CFRD risk.

## Supplementary material

The following online material is available for this article:

Figure S1 **-**
Area under the curve for insulin secretion and insulin secretion normalized by glucose were significantly higher in the F508del/L206W subgroup.
