# Supplementary material for: Association between cystic fibrosis transmembrane regulator genotype and clinical outcomes, glucose homeostasis indices and CF-related diabetes risk in adults with CF
Source: Genet Mol Biol. 2024 Mar 29;47(1):e20230021. doi: 10.1590/1678-4685-GMB-2023-0021 (PMC10993309; doi:10.1590/1678-4685-GMB-2023-0021)
Supplement: Figure S1 - [file 1415-4757-GMB-47-1-e20230021-s1.pdf]

Genetics and Molecular Biology
https://doi.org/10.1590/1678-4685-GMB-2023-0021

Supplementary Material to "Association between Cystic Fibrosis Transmembrane Regulator genotype and clinical outcomes, glucose homeostasis indices and CF-related Diabetes risk in adults with CF"

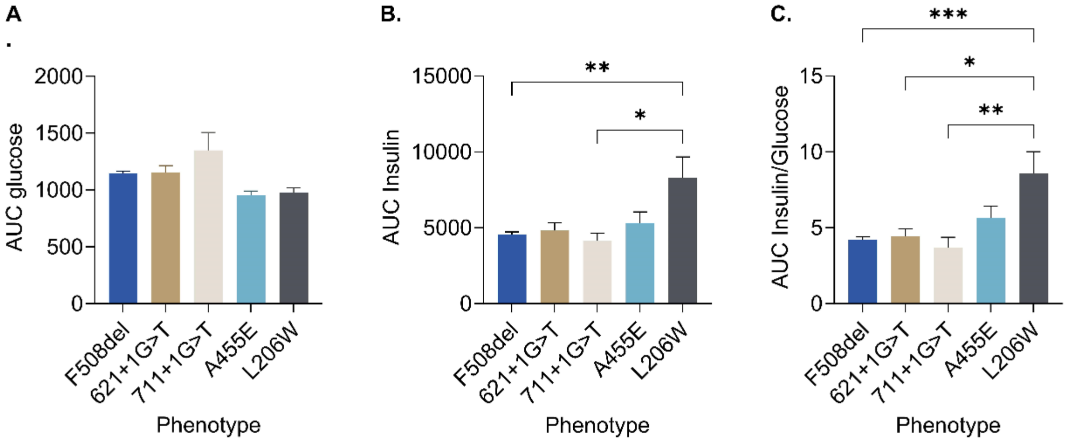

Figure S1 - Area under the curve for insulin secretion and insulin secretion normalized by glucose were significantly higher in the F508del/L206W subgroup. AUC glucose (A), AUC insulin (B), and AUC insulin/glucose (C) based on genotype sub-group. Significance was determined using one-way ANOVAs and is demonstrated as follows: *p≤0.05; **p≤0.01; ***p≤0.0001.
